# Correction of Single-Tooth Crossbite Using a 2x4 Appliance: A Clinical Case Report

**DOI:** 10.7759/cureus.71818

**Published:** 2024-10-18

**Authors:** Gaurav S Yermalkar, Namrata Gaonkar, N.D. Shashikiran, Sachin Gugawad, Savita G Hadakar, Sonali Waghmode, Ankita Maurya

**Affiliations:** 1 Department of Paedodontics and Preventive Dentistry, School of Dental Sciences, Krishna Vishwa Vidyapeeth, Karad, IND

**Keywords:** 2x4 appliance, anterior crossbite, malocclusion of teeth, pediatric dentistry, reverse overjet

## Abstract

The 2x4 appliance is a versatile treatment modality that consists of bands cemented or bonded to the first permanent maxillary molars with brackets bonded to the labial surface of maxillary incisors. A continuous archwire is engaged in the buccal tubes of the molar bands through the brackets of the incisors. This arrangement enables comprehensive control over the anterior teeth, allowing a change in their positioning through precise and efficient movements. The appliance does not require frequent adjustments, is well-accepted by patients, and contributes to expedited treatment outcomes. This case report details the correction of a single-tooth anterior crossbite with the use of a 2x4 appliance. The results not only met the aesthetic expectations of the patient but also addressed the concerns of the parents.

## Introduction

Anterior crossbite refers to an abnormal labiolingual relationship involving one or more anterior maxillary and mandibular incisors. Clinically, it presents as a reverse overjet, where one or more maxillary incisors are positioned lingually in relation to the mandibular incisors during occlusion in centric relation [1]. The etiology of anterior crossbite is multifactorial, with contributing factors classified into skeletal, dental, and functional categories, depending on the underlying nature of the malocclusion [2].

Anterior crossbite can have a detrimental impact on periodontal health, potentially resulting in gingival recession, alveolar bone thinning, and increased mobility of the opposing mandibular teeth. Functional crossbite, arising from premature occlusal contact, may contribute to mandibular deviation and the development of temporomandibular joint dysfunction and pain [3]. A range of treatment modalities are available for the correction of anterior crossbite, including removable appliances with Z springs targeting the maxillary incisors, Hawley appliances with auxiliary springs, inverted labial bows, acrylic inclined planes, Catlan’s appliances, fixed appliances utilizing a multi-bracket system, bonded resin composite slopes, and the 2×4 appliance.

The 2×4 appliance presents several advantages compared to other techniques as it offers precise control over the positioning of anterior teeth, is highly tolerated by patients, requires no patient intervention for adjustments, and facilitates efficient and accurate tooth movement. The appliance is composed of brackets affixed to the maxillary incisors, bands placed on the maxillary first permanent molars, and a continuous archwire connecting these components [4].

This case report focuses on the correction of a single-tooth crossbite using a 2x4 appliance.

## Case presentation

A 13-year-old boy presented at the Department of Pediatric and Preventive Dentistry with a primary concern of misaligned teeth in the upper anterior region. There was no significant family or medical history. Also, there was no history of any habits. Intraoral examination revealed that the patient was in the permanent dentition phase of occlusion and the maxillary right central incisor was positioned palatally, leading to an anterior crossbite. Cephalometric and model analyses were carried out to arrive at the diagnosis of Angle's class 1 malocclusion with anterior crossbite in relation to tooth number: 11 (Figure 1).

**Figure 1:**
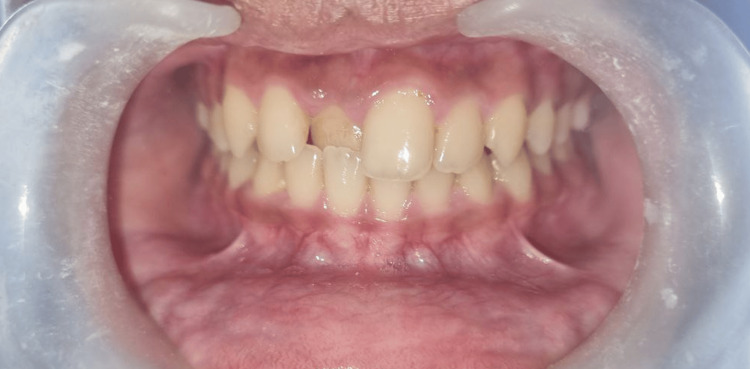
Pre-treatment photograph

Following a thorough discussion of the treatment options with the patient's parents, informed consent was obtained. To initiate treatment, orthodontic molar bands with buccal tubes were cemented on the maxillary first molars bilaterally. Subsequently, metal brackets, MBT system 0.022″ slot, were bonded to the labial surfaces of the four maxillary permanent incisors.

A 0.014″ round nickel-titanium (Ni-Ti) archwire was inserted into the bracket slots and engaged in the molar tubes bilaterally. Glass ionomer cement (GIC) (3M Ketac Cem) buildup of 2 mm was used as posterior bite ramps on the mandibular permanent molars bilaterally. GIC is ideal for creating a bite ramp due to its temporary nature and ease of removal once the treatment is finished. This was done to disocclude the occlusion and thereby achieve a 2 mm incisal clearance. The archwire was stabilized in position using elastic modules and the patient was recalled after one month since ideally tooth movement is achieved within 4-5 weeks (Figure 2). 

**Figure 2:**
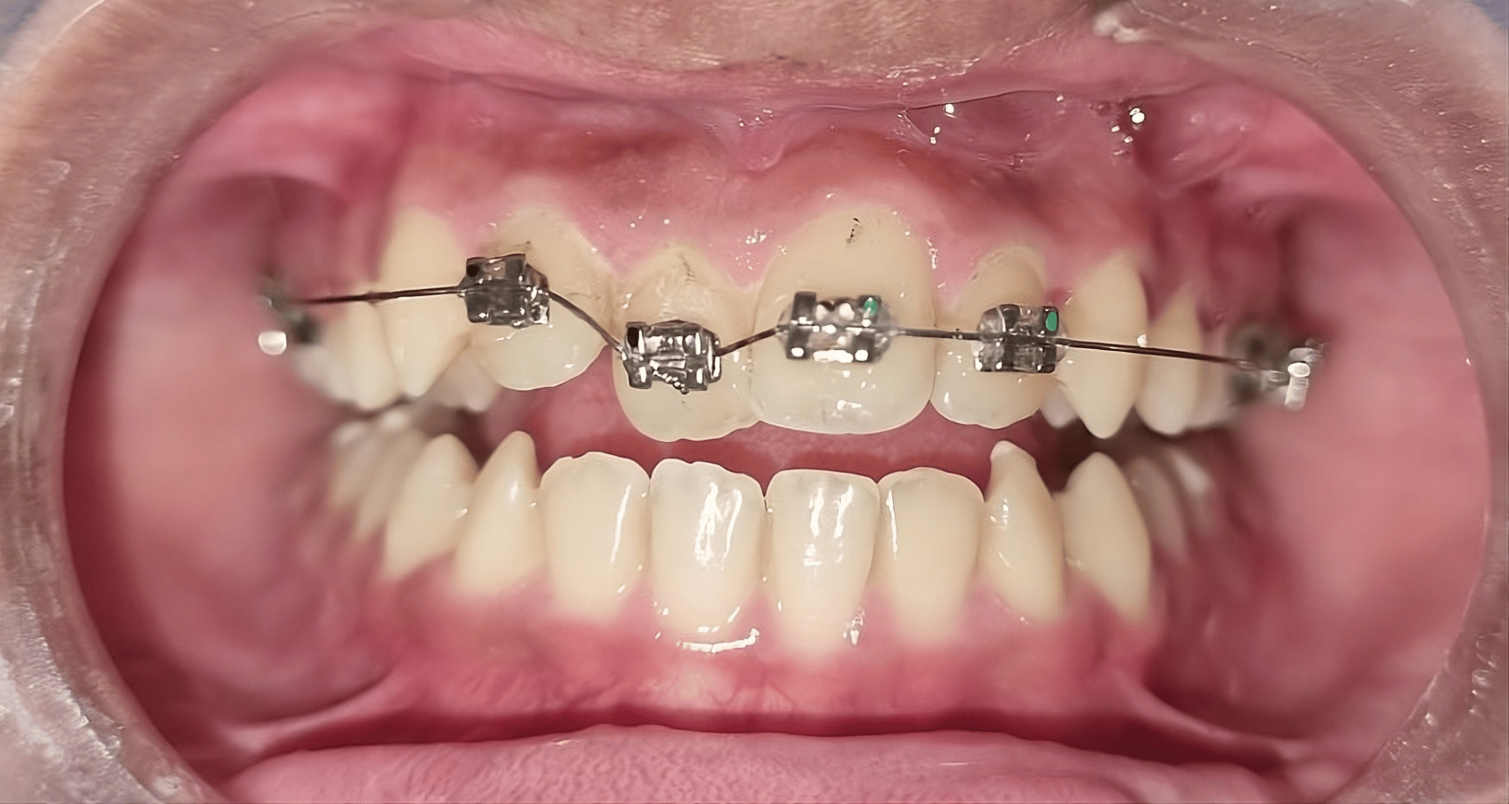
Placement of the nickel-titanium (Ni-Ti) 0.014″ round archwire

At the next appointment, the 0.014″ round Ni-Ti archwire was replaced with a 0.016″ one (Figure 3).

**Figure 3:**
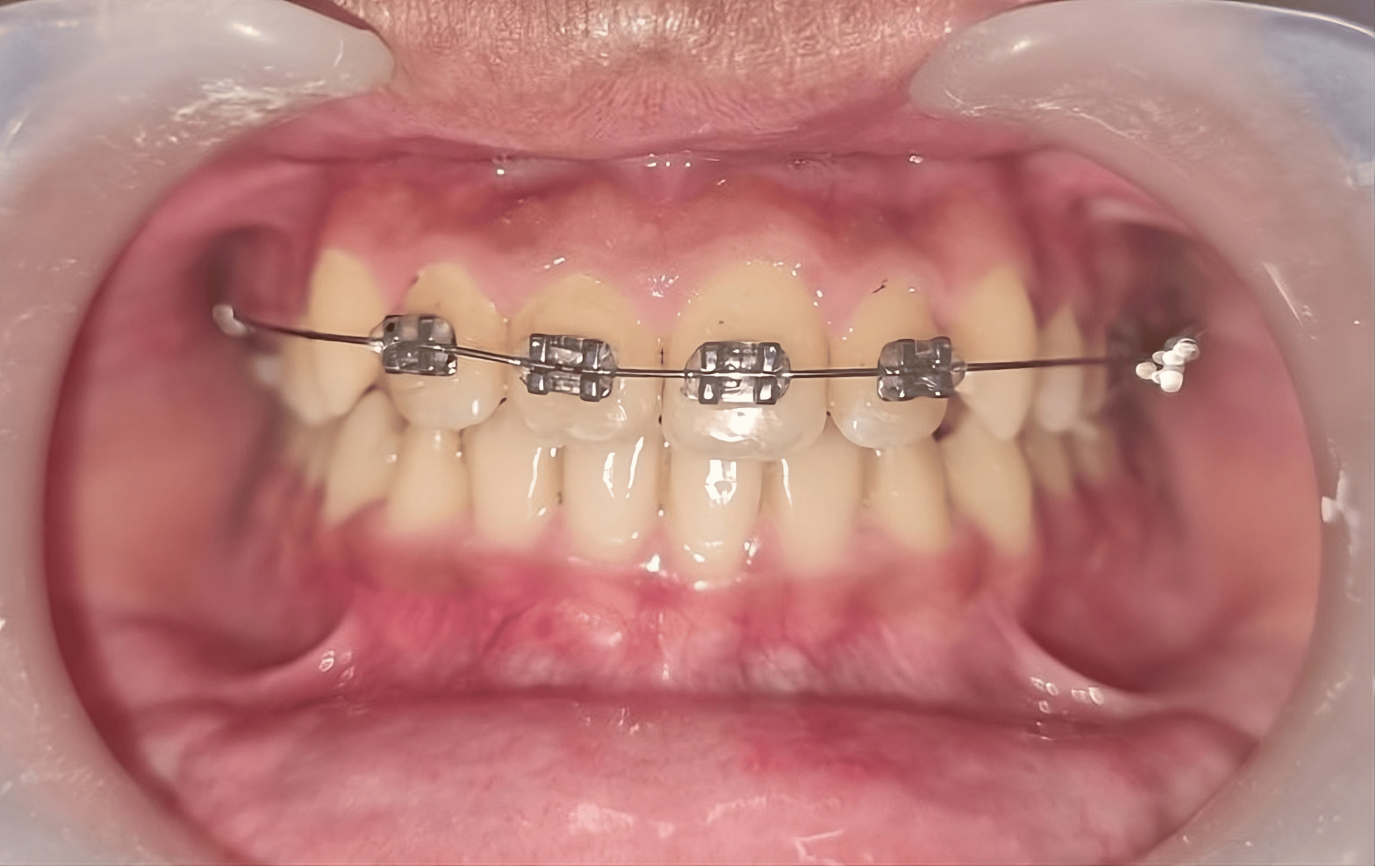
Placement of the nickel-titanium (Ni-Ti) 0.016″ round archwire

After a period of one month, the 0.016″ round Ni-Ti archwire was changed to 0.016 x 0.022’’ Ni-Ti rectangular wire and retained for another month before the brackets were debonded (Figure 4).

**Figure 4:**
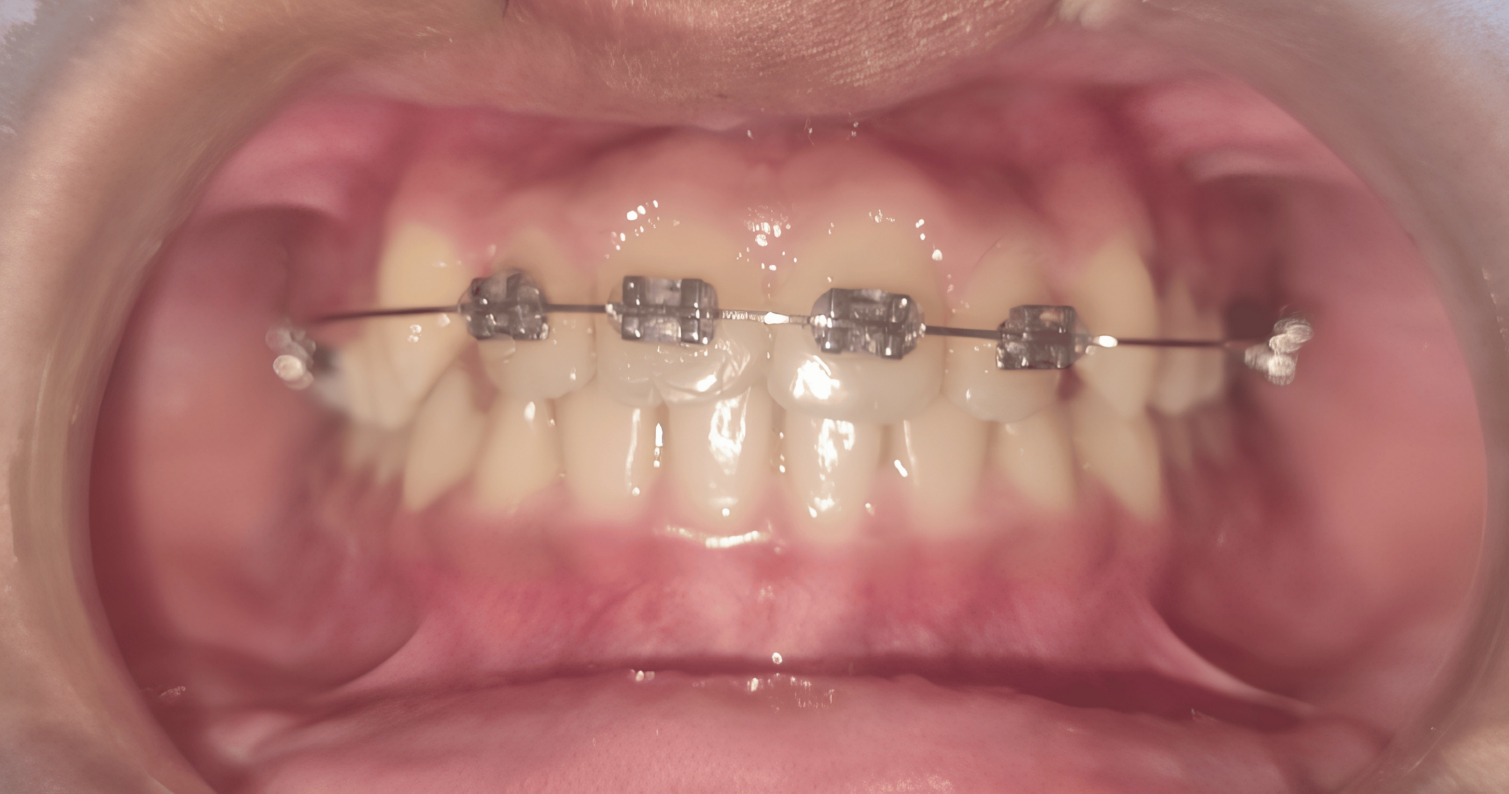
Placement of the nickel-titanium (Ni-Ti) 0.016 x 0.022’’ rectangular wire archwire

After debonding, the anterior crossbite was successfully resolved with the four maxillary incisors aligned in their correct positions without any disruption to the occlusion (Figure 5). 

**Figure 5:**
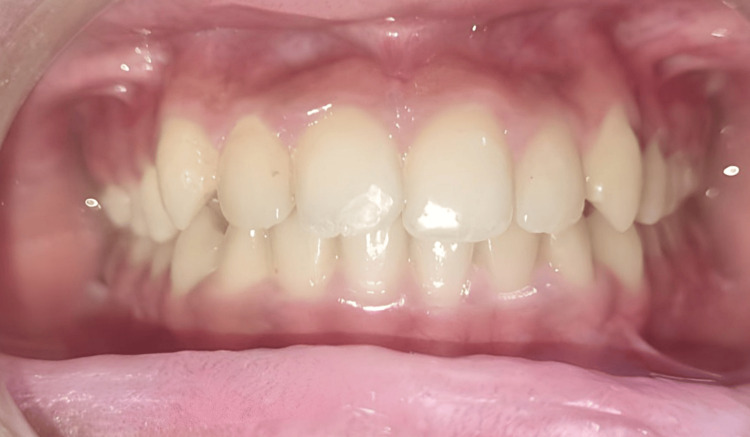
Post-treatment photograph

No retainer was required after the correction of crossbite as a single-tooth anterior crossbite is self retentive. The results not only met the aesthetic expectations of the patient but also satisfied the concerns of the parents. The patient has been asked to follow up after six months.

## Discussion

Removable appliances have been proposed as an effective approach for correcting anterior tooth malposition, resolving teeth in lingual crossbite, and addressing constricted maxillary arches [5]. Removable appliances present certain limitations, primarily due to their limited control over tooth positioning. They typically apply single-point forces, often resulting in simple tipping movements rather than precise tooth movement. Additionally, patients may frequently remove and reinsert the appliance, leading to stress fractures in the retaining cribs or clasps, compromising retention. This loss of retention can result in reduced compliance, as patients may be inclined to discontinue use. Ninou and Stephens highlighted patient cooperation and appliance retention as the primary challenges associated with the use of removable expansion appliances [6].

The aforementioned challenges associated with removable appliances can be effectively addressed by utilizing an alternative approach, such as the 2×4 appliance. As a sectional fixed appliance, the 2×4 system allows for a more efficient and precise tooth movement, offering three-dimensional control during the correction of malaligned anterior teeth. This results in enhanced treatment outcomes compared to removable options [1]. Consequently, diastemas, rotations, and improper inclinations of teeth can be effectively and efficiently addressed using this technique [7,8]. It includes bonded brackets on the permanent maxillary incisors, bands on the first permanent maxillary molars, and a continuous archwire. Lee et al. identified the following factors which should be considered before selecting a treatment approach: the presence of adequate space in the dental arch for the repositioning of the tooth, sufficient overbite to maintain the corrected position, and an apical position of the tooth in crossbite that aligns with its position in Class I occlusion [1].

The advantages of using the 2×4 appliance include minimal reliance on patient cooperation, reduced treatment duration, absence of laboratory costs, and the ability to facilitate versatile orthodontic tooth movements and corrections. MBT brackets are incorporated with ideal tip and torque values ensuring that the teeth are placed in the correct axial inclination. However, some disadvantages include the risk of dangling wires during eating and brushing, particularly in pediatric patients, as well as the potential for plaque accumulation around the brackets and bands. Nonetheless, these challenges can be effectively managed with proper oral hygiene practices [9].

## Conclusions

In the presented case report, the 2×4 appliance proved to be a highly versatile and effective tool for correcting single-tooth crossbite. Its practical design and impactful results make it a valuable treatment option. The appliance's simplicity, combined with its capacity to address both functional and aesthetic issues, establishes it as a superior alternative to traditional methods for managing these complex orthodontic challenges.
